# Adult mice are unresponsive to AAV8-Gremlin1 gene therapy targeting the liver

**DOI:** 10.1371/journal.pone.0247300

**Published:** 2021-02-19

**Authors:** Roxana Khatib Shahidi, Jenny M. Hoffmann, Shahram Hedjazifar, Laurianne Bonnet, Ritesh K. Baboota, Stephanie Heasman, Christopher Church, Ivet Elias, Fatima Bosch, Jeremie Boucher, Ann Hammarstedt, Ulf Smith

**Affiliations:** 1 The Lundberg Laboratory for Diabetes Research, Department of Molecular and Clinical Medicine, the Sahlgrenska Academy, University of Gothenburg, Gothenburg, Sweden; 2 Wallenberg Center for Molecular and Translational Medicine, University of Gothenburg, Gothenburg, Sweden; 3 Bioscience Metabolism, Research and Early Development, Cardiovascular, Renal and Metabolism (CVRM), BioPharmaceuticals R&D, AstraZeneca, Cambridge, United Kingdom; 4 Center of Animal Biotechnology and Gene Therapy and Department of Biochemistry and Molecular Biology, School of Veterinary Medicine, Universitat Autònoma de Barcelona, Bellaterra and Centro de Investigación Biomédica en Red de Diabetes y Enfermedades Metabólicas Asociadas (CIBERDEM); 5 Bioscience Metabolism, Research and Early Development, Cardiovascular, Renal and Metabolism (CVRM), BioPharmaceuticals R&D, AstraZeneca, Gothenburg, Sweden; Medical University of Vienna, AUSTRIA

## Abstract

**Objective:**

Gremlin 1 (GREM1) is a secreted BMP2/4 inhibitor which regulates commitment and differentiation of human adipose precursor cells and prevents the browning effect of BMP4. GREM1 is an insulin antagonist and serum levels are high in type 2 diabetes (T2D). We here examined in vivo effects of AAV8 (Adeno-Associated Viral vectors of serotype eight) *GREM 1* targeting the liver in mature mice to increase its systemic secretion and also, in a separate study, injected recombinant GREM 1 intraperitoneally. The objective was to characterize systemic effects of GREM 1 on insulin sensitivity, glucose tolerance, body weight, adipose cell browning and other local tissue effects.

**Methods:**

Adult mice were injected with AAV8 vectors expressing GREM1 in the liver or receiving regular intra-peritoneal injections of recombinant GREM1 protein. The mice were fed with a low fat or high fat diet (HFD) and followed over time.

**Results:**

Liver-targeted AAV8-GREM1 did not alter body weight, whole-body glucose and insulin tolerance, or adipose tissue gene expression. Although GREM1 protein accumulated in liver cells, GREM1 serum levels were not increased suggesting that it may not have been normally processed for secretion. Hepatic lipid accumulation, inflammation and fibrosis were also not changed. Repeated intraperitoneal rec-GREM1 injections for 5 weeks were also without effects on body weight and insulin sensitivity. UCP1 was slightly but significantly reduced in both white and brown adipose tissue but this was not of sufficient magnitude to alter body weight. We validated that recombinant GREM1 inhibited BMP4-induced pSMAD1/5/9 in murine cells in vitro, but saw no direct inhibitory effect on insulin signalling and pAkt (ser 473 and thr 308) activation.

**Conclusion:**

GREM1 accumulates intracellularly when overexpressed in the liver cells of mature mice and is apparently not normally processed/secreted. However, also repeated intraperitoneal injections were without effects on body weight and insulin sensitivity and adipose tissue UCP1 levels were only marginally reduced. These results suggest that mature mice do not readily respond to GREMLIN 1 but treatment of murine cells with GREMLIN 1 protein in vitro validated its inhibitory effect on BMP4 signalling while insulin signalling was not altered.

## 1. Introduction

Bone Morphogenetic Proteins (BMP) are important regulators of embryogenesis and their signalling has to be carefully regulated. GREMLIN (GREM) 1 and 2 are members of the DAN family and regulate BMP signalling with a careful temporal-spatial gradient to allow normal development of limbs, kidneys and lungs [1, 2]. GREM 1 knockout is lethal and leads to kidney agenesis [3–5]. Early studies indicated that GREM 1 is also pro-angiogenic by binding to VEGFR2 [6] but the VEGFR2 target could not be reproduced in a recent extensive study [7]. GREM 1 is also profibrotic in kidneys and lungs and it is increased in diabetic kidney disease [3]. In addition to its regulatory effects on organ development and cell fibrosis, we found GREM 1 to be highly expressed in human adipose tissue and secreted by the adipose cells [8, 9]. Furthermore, it is antagonistic to insulin in human target cells, increased in serum in obesity and, most strongly, in Type 2 diabetes and also upregulated in human liver biopsies from individuals with NAFLD/NASH [10]. Thus, GREMLIN 1 seems to be quite a pleiotrophic molecule and circulating hormone.

Obesity and associated metabolic and health consequences are increasing at an epidemic rate worldwide. The ability to recruit new adipose cells to store excess lipids is protective of the metabolic complications of obesity while hypertrophic expansion of adipocytes promotes inflammation and insulin resistance [11]. Commitment of adipose tissue mesenchymal precursor cells into the white adipose lineage is regulated by BMP4 [8, 9, 12] which also is secreted by adipose cells and increased as the adipose cells expand [9]. BMP4 signalling is regulated by several endogenous BMP antagonists, including GREMLIN 1 and NOGGIN, both of which are also expressed in adipose tissue cells [13, 14]. Interestingly, we have shown that inhibiting GREM 1 in the adipose tissue and, thus, enhancing cell BMP4 action, promotes adipogenesis and human adipose precursor cells to differentiate towards a beige/brown phenotype with increased mitochondrial biogenesis and oxidative capacity [9]. Increased browning of the white adipose cells was also seen in transgenic mice overexpressing BMP4 in the adipose tissue [15]. Furthermore, increasing serum BMP4 levels in adult mice through gene therapy targeting the liver with AAV8 BMP4 promotes white adipose tissue browning, increases whole-body energy expenditure and prevents diet-induced obesity in initially lean mice [16]. Similar BMP4 gene therapy in adult and initially obese mice showed that whole-body insulin sensitivity was increased, insulin signalling in target tissues enhanced but, in initially obese mice, body weight remained unchanged and there was no change in energy-expenditure or browning of the adipose tissue, most likely due to local adipose tissue BMP4 resistance by increased NOGGIN levels [17]. Thus, reducing adipose tissue endogenous BMP inhibitors to enhance BMP4 action represents an attractive possibility to prevent obesity and increase insulin sensitivity while, in contrast, increasing these antagonists would reduce adipose tissue browning, enhance obesity and associated metabolic complications.

We here tested the effect of GREM 1 in vivo in adult mice through two means; enhancing liver expression and subsequent secretion of GREM 1 to the bloodstream following AAV8-GREM1 gene therapy and, by repeated injections of recombinant GREM 1 intraperitoneally in adult mice. Surprisingly, we found AAV8-produced GREM 1 protein to be accumulated in liver cells without any negative effects on liver inflammation, fibrosis or whole-body metabolism. However, it was apparently not correctly processed by the liver cells to be cleaved and secreted as also circulating levels were unchanged. An important caveat for this conclusion relates to the sensitivity of the assay system which may not have been able to pick up small changes in the circulating levels. We, therefore, also used another approach but unexpectedly also repeated intraperitoneal injections of GREM 1 did not lead to any significant changes in body weight, inflammation or insulin sensitivity and only exerted trivial effect on adipose tissue UCP1 protein. These negative effects could possibly also be due to protein binding and accumulation of GREM1 in the liver cells since the viscera is drained by the portal vein system. Together, our data show that mature mice are essentially unresponsive to the BMP4 antagonist GREM 1 at least when administered through targeting the liver. Interestingly, *GREM* 1 mRNA levels are not increased, but actually reduced, in the adipose tissue in obese mice [10, 16] in contrast to what is seen in human adipose tissue in obesity [8, 10]. Instead, NOGGIN is increased in obese mice [16]. Thus, these findings suggest species specificity in tissue regulation of BMP2/4 signaling and action which also is supported by the present findings.

## 2. Materials and methods

### 2.1. Ethics statement

All animal experiments were performed after prior approval from the Ethics Committee for Animal Studies at the administrative Court of Appeal in Gothenburg, Sweden.

### 2.2. Animal models

Male C57BL6/N (Taconic, Hudson, NY, USA) mice were housed 4 per cage and maintained on a 12h light-dark cycle in a temperature (21°C) and humidity-controlled facility with free access to water and food underwent weekly weighing. For the first 12 weeks of the study, the mice (cohort 1 and 2) were fed a control diet (10 kcal% fat, Research Diets, New Brunswick, NJ, USA). Cohort 1 (used for AAV-8 injection) was fed a 45 kcal% fat high-fat diet (HFD, Research Diets) from week 12 until termination at study week 30. Cohort 2 mice were fed 60 kcal% HFD (Research Diets) for 26 weeks and thereafter received intraperitoneal injections (IP) of mouse rec-GREM 1 (0.8μg/g mice; R&D Systems, Minneapolis, USA) or equal volume of 2xDPBS, 1mM EDTA, pH 6.8 twice per week for a total of 5 weeks. At termination, blood was collected by heart puncture and serum was stored immediately at -80°C. Tissues were weighed and collected for morphological analysis or snap frozen in liquid nitrogen and stored at -80°C for analysis of protein and gene expression.

### 2.3. Recombinant Adeno-Associated Viral (AAV) vectors

Recombinant AAV vectors of serotype eight encoding a codon-optimized mouse *GREM 1* cDNA sequence under control of the human Alpha 1-Antitrypsin (hAAT1) promoter were produced by triple transfection of HEK293 cells and followed by an optimized caesium chloride gradient-based purification. A non-coding plasmid carrying the hAAT1 promoter was used to produce empty vectors for control mice. The AAV *GREM 1* cDNA expressed a full-length protein including the secretion sequence.

### 2.4. In vivo administration of AAV vectors

AAV vectors (5 x10 ^11^ viral particles/200 μL saline/mouse) (9 mg/mL saline; Fresenius Kabi, Bad Homburg, Germany), were administered via tail vein injection in 6-week-old mice, at study week 0. The mice were started on study control diet concurrently with the AAV injections.

### 2.5. Glucose Tolerance Test (GTT), Insulin Tolerance Test (ITT) and Pyruvate Tolerance Test (PTT)

Following 4h of food withdrawal at time 0, mice received intraperitoneal injections with glucose (1 g/kg; Fresenius Kabi, Bad Homburg, Germany) or human recombinant insulin (1.3 U/kg, cohort 1 and 1.2 U/kg cohort 2; Actrapid Penfill; Novo Nordisk, Bagsvard, Denmark) or pyruvate (2g/kg; Sigma-Aldrich, Saint Louis, MO, USA) for GTT, ITT and PTT, respectively. Blood samples were collected from the tail vein and glucose levels were measured at time 0 (basal), 15, 30, 60, 90- and 120-min post-injection by Accu-Chek glucometer (Roche Diagnostics, Basel, Switzerland).

### 2.6. Biochemical assays

Insulin levels (fasting and during GTT) were measured using Insulin ELISA kit (Ultrasensitive Mouse Insulin ELISA Kit #90080, Chrystal Chem Inc., Downers Grove, IL, USA). Serum amyloid A isoform 3 (SAA-3) was measured with a SAA-3 ELISA kit (#EZMSAA3- 12K, Merck Millipore, Denmark, Germany). In-house GREM 1 serum assay in human samples has been reported previously using the MedImmune-produced antibody (Med Immune Astra Zeneca, Gaithersburg, MD) [9].

### 2.7. Ex vivo isolation of mouse adipocytes

Adult mouse adipocytes were isolated and processed as previously described [16, 17]. Briefly, the adipose tissue was digested with Collagenase type II (Sigma-Aldrich, St. Louis, MO, USA) for 60 min at 37°C in a shaking water bath. The adipocytes were then filtered and washed, followed by cell size measurement.

### 2.8. Histology and immunohistochemistry

Tissues were fixed in 4% formaldehyde (Histolab Product AB, Gothenburg, Sweden) and maintained in 70% ethanol, embedded in paraffin or snap frozen in optimal cutting temperature (OCT) mounting medium (Histolab) and maintained at -80°C for later sectioning.

Liver, subcutaneous white adipose tissue (SubQ WAT) and kidney were paraffin sectioned and stained with Hematoxylin and Eosin. For immunohistochemical staining of liver and SubQ WAT, primary antibodies against F4/80 (1:200, ab111101, AbCam), Ly6C (1:300, ab 15627, AbCam) and UCP1 (1:1000, ab10983, AbCam) were used followed by biotinylated anti-goat secondary antibody (E0466; Dako, Glostrup, Denmark) and horseradish peroxidase-conjugated streptavidin (P0397; Dako). DAB was used for visualisation and sections were counterstained with Hematoxylin Eosin. Additional staining with Masson trichrome and picrosirious red was done on paraffin sections from liver. Kidney periodic acid staining (PAS) was performed on paraffin sections (Histolab, manufacturer protocol). Oil Red O staining was performed on cryosectioned liver tissue. Quantification was performed using images from 10 randomly selected 10x or 20x microscope field per mouse using imageJ version 1.47 software (National Institutes of Health, Bethesda, MD). Liver sections were stained for GREM 1 using a specific antibody (generated by MedImmune/Astra Zeneca, Gaithersburg, MD, USA) [9].

### 2.9. Analysis of hepatic and kidney collagen

To determine collagen content, liver and kidney samples were homogenised in dH2O, hydrolysed in 12N HCL for 3h at 120°C and analysed using a hydroxyproline colorimetric assay kit (#MAK008, Sigma-Aldrich, Saint Louis, MO, USA).

### 2.10. Quantitative real time PCR

RNA was isolated with the RNEasy Mini Kit (Qiagen, Valencia, CA, USA). cDNA was produced using the High Capacity cDNA Reverse Transcription Kit (Applied Biosystems, Foster City, CA, USA) and reverse transcriptase PCR. Gene-specific primers and probes were designed using the software Primer Express or purchased on-demand (Applied Biosystems, Carlsbad, CA, USA). Target transcripts were quantified from duplicate samples after normalisation of the data against Eukaryotic 18S rRNA (Applied Biosystem) as endogenous control.

### 2.11. Western blot analysis

Immunoblotting was performed as described [14] with the following commercial primary antibodies: SMAD 1/5/9 (ab66737, Abcam, Cambridge, UK), total OXPHOS Antibody Cocktail (ab 110413, Abcam, Cambridge, UK), UCP1 (MAB6158, R&D System), β-tubulin (#2128), pSMAD 1/5/9 (#13820), Akt (#9272) and phospho-Akt (ser 473) (#9271) (all from Cell Signalling Technology, Danvers, MA, USA) and phospho-Akt (thr308) (#9275S BioLabs). Quantifications were performed by normalisation against loading controls.

### 2.12. In vitro cell culture

Immortalised pre-adipocyte mouse cell line (3T3/L1) was differentiated into adipocytes by incubation for 48h in Dulbecco’s minimal Eagle’s medium (DMEM) containing 10% FCS, 1% Pest, 1% Glutamine containing 1 *μ*M dexamethasone, 0.5 mM isobutylmethylxanthine (IBMX) and 100 nM human recombinant insulin (Actrapid Penfill; Novo Nordisk, Bagsvard, Denmark), followed by 48h 100nM insulin in growth media (DMEM containing 10% FCS, 1% Pest, 1% Glutamine) and then for 48h in growth media. Insulin stimulation was performed after 4h serum starvation in DMEM media containing 1% Pest, 0.5% BSA, 0.1% Glutamine, in addition to treatment with rGREM 1 or rBMP4 (#956-GR and #5020-PB, respectively, R&D System), or both, followed by 10 min incubation with 10 or 100nM insulin. We also obtained rGREM 1 with verified correct protein folding from Novo Nordisk, Copenhagen, Denmark (a kind gift by Dr Morten Tonnesen) which was used for control experiments in the insulin signalling studies as shown.

Immortalised mouse myoblast cell line (C2C12) was differentiated into myotubes by incubating the cells with 2% horse serum in high glucose and high glutamine DMEM (Gibco, Paisler, UK) for 7 days. Insulin stimulation was performed similar to the protocol above in starvation media (DMEM containing 1% Pest, 0.1% Glutamine).

### 2.13. Statistical analysis

Statistics were performed using Excel (Microsoft office, Redmond, WA, USA). Statistical significance between two groups was calculated with an unpaired two-tailed Student’s *t* Test or, when comparing several groups, with ANOVA. A p-value <0.05 was considered statistically significant.

## 3. Results

### 3.1. Increased GREM1 accumulation in the liver following AAV8-GREM1 gene treatment

**Fig 1:**
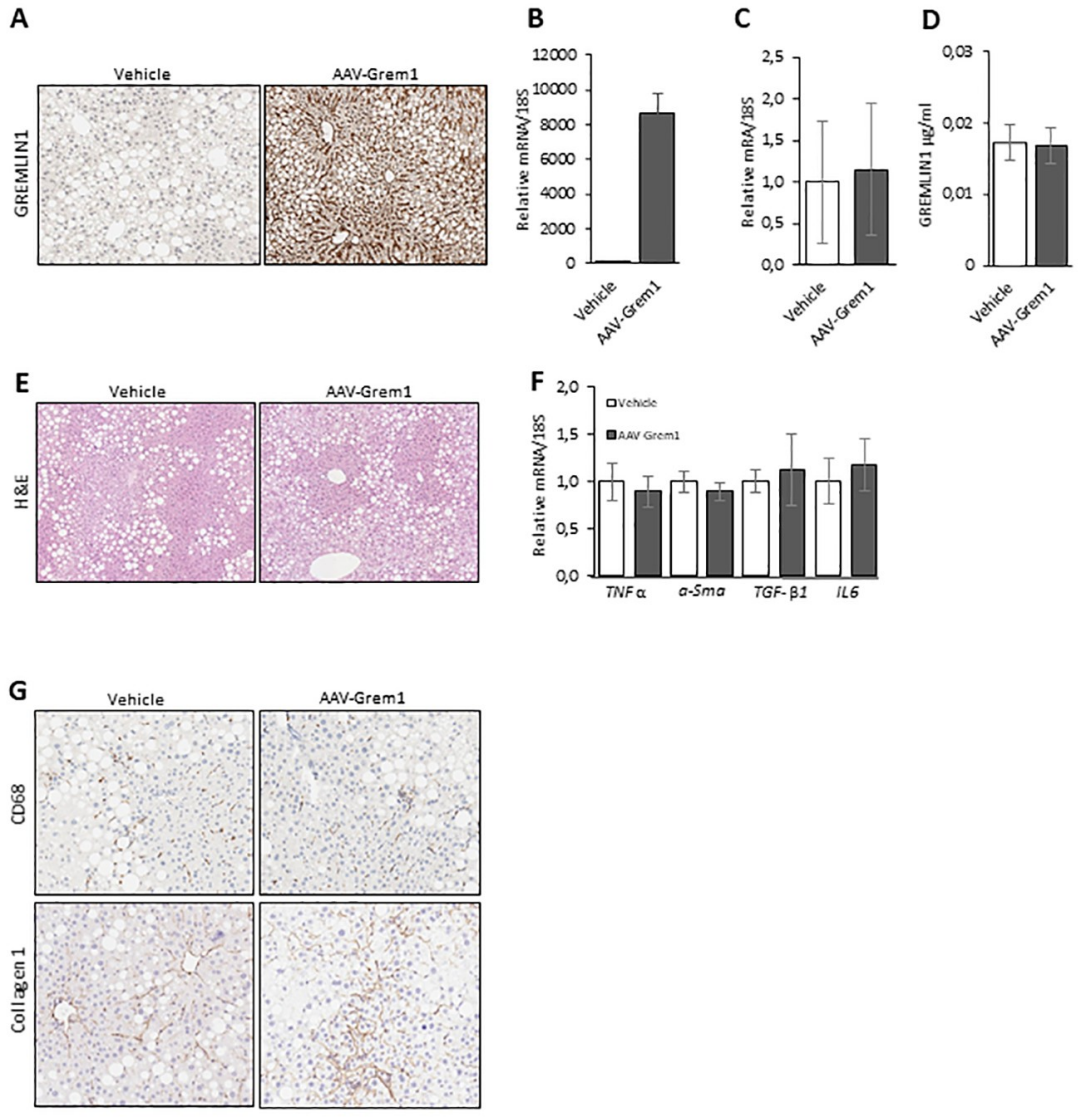
Effect of AAV8-Gremlin 1 gene transfer on liver and BAT in 30 weeks old male mice (C57BL6/N) after 12 weeks of LFD followed by 18 weeks of HFD. (A) Representative light microscopy image X10 from liver sections showing hepatic Gremlin 1 protein expression (n = 8 in each group). (B) qRT-PCR of AAV8-Gremlin1 mRNA/18S in liver (optimized sequence) and (C) endogenous Gremlin 1 in BAT. (D) Gremlin 1 levels in serum. (E) Representative light microscopy image X10 from liver sections showing no difference in lipid accumulation (n = 8 in each group). (F) qRT-PCR of inflammation and fibrosis markers (mRNA/18S). (G) Representative light microscopy image X20 from liver sections showing hepatic CD68 and Collagen 1 (n = 8 each group).

Endogenous liver *GREM1* mRNA levels were extremely low/undetectable in the untreated mice. The combination of AAV vectors of serum type 8 and the hAAT promoter is validated to ensure liver-specific transgene expression from two weeks after injection throughout the life span of a mouse [13, 18, 19] and has successfully been used for BMP4 gene treatment in our laboratory [16, 17]. As expected, hepatic GREM1 protein expression was increased in AAV8-GREM1 compared to AAV8-vehicle control mice (Fig 1A) and optimized AAV8-*GREM1* mRNA levels were also high (Fig 1B). In contrast, endogenous *GREM1* mRNA levels in WAT (very low detection level) and BAT were unchanged in both animal models (Fig 1C).

To confirm that GREM1 was secreted to the circulation, we first attempted to measure GREM1 in serum samples with commercially available ELISA kits. Unfortunately, none of the antibodies/kits used were sufficiently sensitive to measure low relevant concentrations of recombinant GREM1 protein. However, using a modified in-house ELISA [9], we were able to detect GREM1 in serum, but the levels were not changed in AAV8-GREM1 mice (Fig 1D) even though the liver clearly expressed high levels of GREM1 (Fig 1A). These data suggest that GREM1 protein is not processed/cleaved and secreted and remains in the liver cells. We also validated that there was no difference in hepatic endogenous *BMP4* and *NOGGIN* expression in the two groups.

### 3.2. No local effects in the liver following AAV8-*GREM1* treatment

We examined if the robust overexpression of GREM1 observed in the liver of AAV8-GREM1 mice led to any negative local effects on hepatic lipid accumulation, inflammation and fibrosis. Liver sections stained for lipid accumulation revealed no difference between groups (Fig 1E). There was also no change in gene expression levels of TNFa, IL-6, a-SMA and TGF-b1 (Fig 1F), or CD68 and Collagen 1 staining (Fig 1G). Together these results show that GREM1 overexpression in the liver of AAV8-GREM1 mice did not increase hepatic lipid accumulation, inflammation or fibrosis.

### 3.3. AAV8-GREM1 treatment did not affect body weight or glucose metabolism

**Fig 2:**
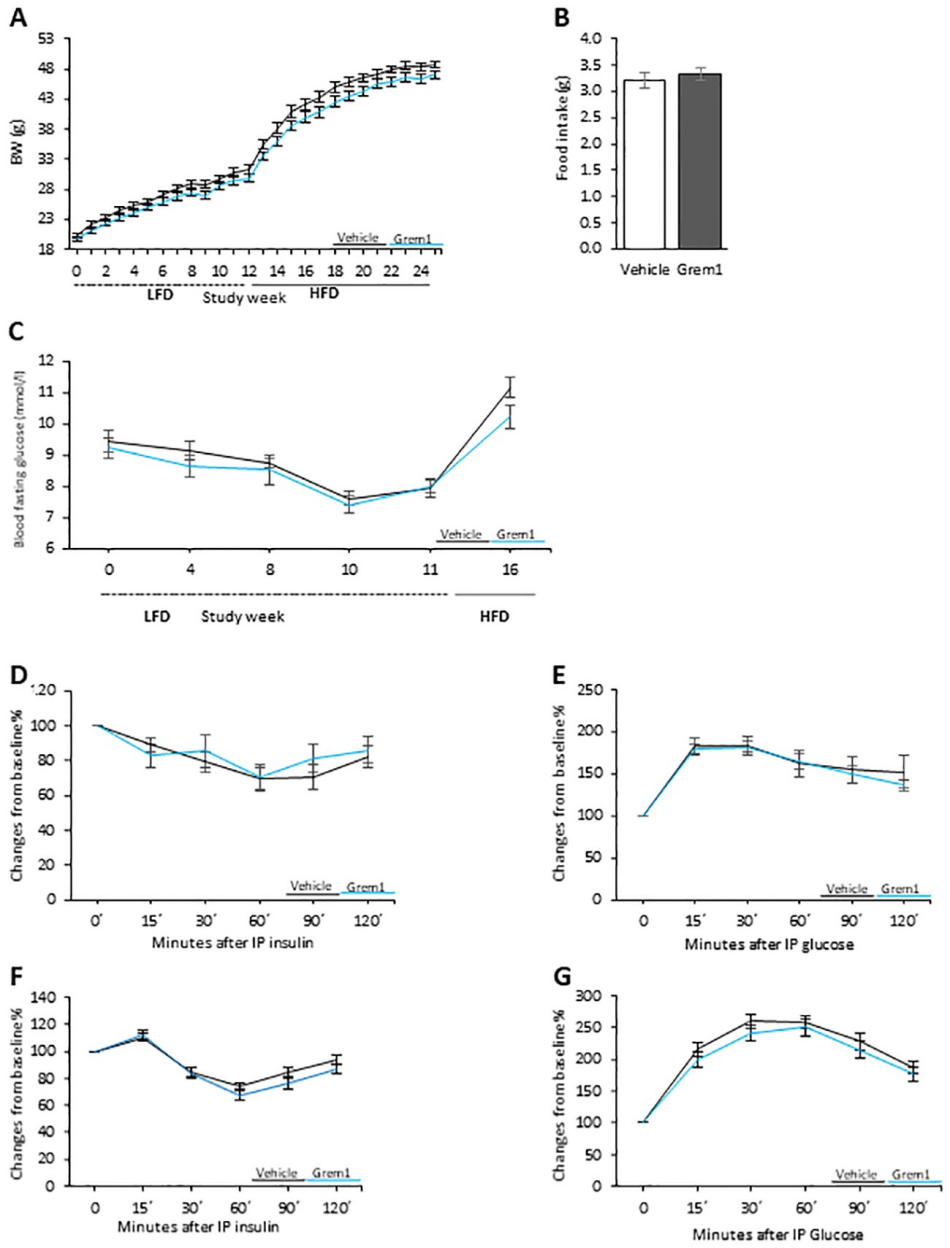
AAV8-Gremlin 1 mice on Low Fat Diet (LFD) or High Fat Diet (HFD) were similar to the control group and hepatic overexpression of Gremlin 1 did not alter metabolic response. Male mice (C57BL6/N) were fed 12 weeks of LFD followed by 18 weeks of HFD, terminated at the age of 30 weeks. (A) Body weights week 1–12 (LFD) and weeks 12–24 (HFD), (B) food intake on LFD or (C) fasting glucose levels. (D) Insulin sensitivity test (ITT, LFD), (E) glucose tolerance test (GTT, LFD), (F) HFD ITT and (G) HFD GTT. Results are means ± S.E.M. n = 12 in each group.

AAV8-vehicle control and AAV8-GREM1 mice were initially fed a normal chow diet for 12 weeks to evaluate the effect of GREM1 on whole body glucose metabolism in the non-obese state. As an inhibitor of BMP4, which enhances browning of white adipose tissue and increases energy expenditure in lean mice [16], we postulated that AAV8-GREM1 would lead to reduced adipose tissue browning, increased body weight and reduced insulin sensitivity. However, there were no differences between AAV8-GREM1 and control mice in body weight, food intake or fasting glucose (Fig 2A–2C). We also did not see any differences in insulin or glucose tolerance between the groups (Fig 2D and 2E). These negative results are consistent with the inability of the liver to secrete GREM1 to the blood.

After the initial period on chow diet, we challenged the mice with a high fat diet (HFD) for an additional 12 weeks to induce severe obesity, which increases adipose tissue cell size and BMP4 levels [9, 11]. However, we did not detect any differences between the groups in terms of body weight (Fig 2A), glucose and insulin tolerance tests (Fig 2F and 2G) under these conditions either. UCP1 expression in white or brown adipose tissue was low and not changed.

Taken together, these data show that GREM 1 was induced in the liver by the AAV8-GREM1 treatment but, within the limitations of our assay system, the protein apparently remained in the liver cells rather than being released to the blood stream which could account for the lack of effects on metabolism and body weight. We, therefore, initiated a second set of experiments with repeated injections of recombinant GREM 1 intraperitoneally. Repeated intraperitoneal injections of GREMLIN 2 in mice have previously been shown to have effects on phenotype [2].

### 3.4. No effect of long-term treatment with recombinant GREM1 protein injections on body weight or insulin sensitivity in HFD mice

**Fig 3:**
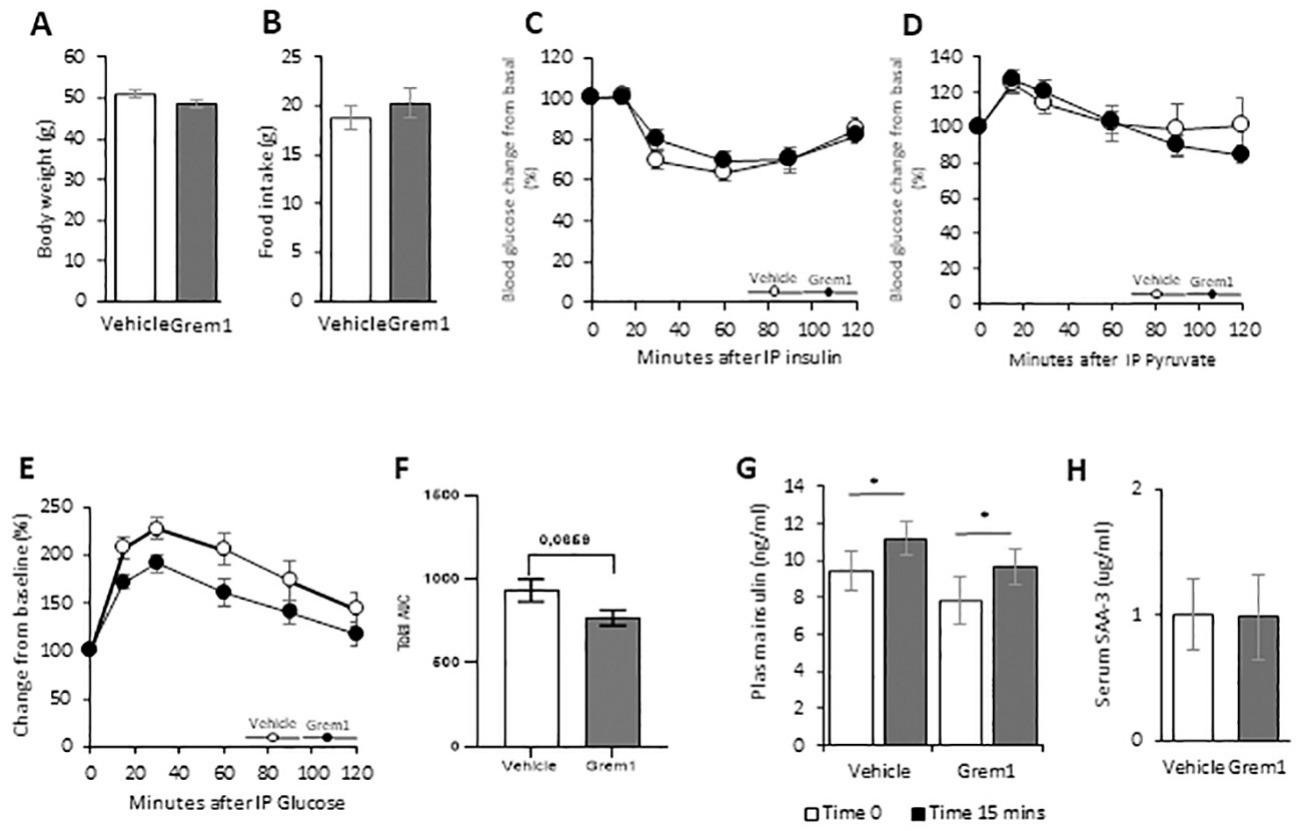
Gremlin 1 IP-injections slightly improved glucose tolerance but had no effect on insulin sensitivity, pyruvate tolerance or whole-body inflammation in 38 weeks old male mice (C57BL6/N) fed 60% HFD. (A) body weight, (B) food intake, (C) insulin sensitivity test (ITT), (D) hepatic gluconeogenesis measured as pyruvate tolerance test (PTT) (E) glucose tolerance test (GTT), (F) total area under the curve (AUC) and (G) insulin levels at 15 min of GTT. (H) Inflammation marker (SAA-3). Results are means ± S.E.M. n = 8 in each group. Statistical significance was determined using Student’s *t* test (two-tailed) or ANOVA as appropriate. *P<0.05.

We injected obese mice with recombinant GREM1 (rGREM1) protein intraperitoneally twice per week for 5 weeks. These repeated rGREM1 injections did not lead to a significant change in body weight (Fig 3A) and there was no difference in food intake (Fig 3B) or in the insulin and pyruvate tolerance tests between the two groups (Fig 3C and 3D). Glucose tolerance was not significantly altered in treated mice, also when analysed as AUC and glucose-stimulated insulin levels measured at 15 min, were also unchanged (Fig 3E–3G). There were no indications of increased inflammation as shown by similar circulating SAA3 levels (Fig 3H).

### 3.5. Minor effects of long-term injections with recombinant GREM1 protein in white and brown adipose tissue

**Fig 4:**
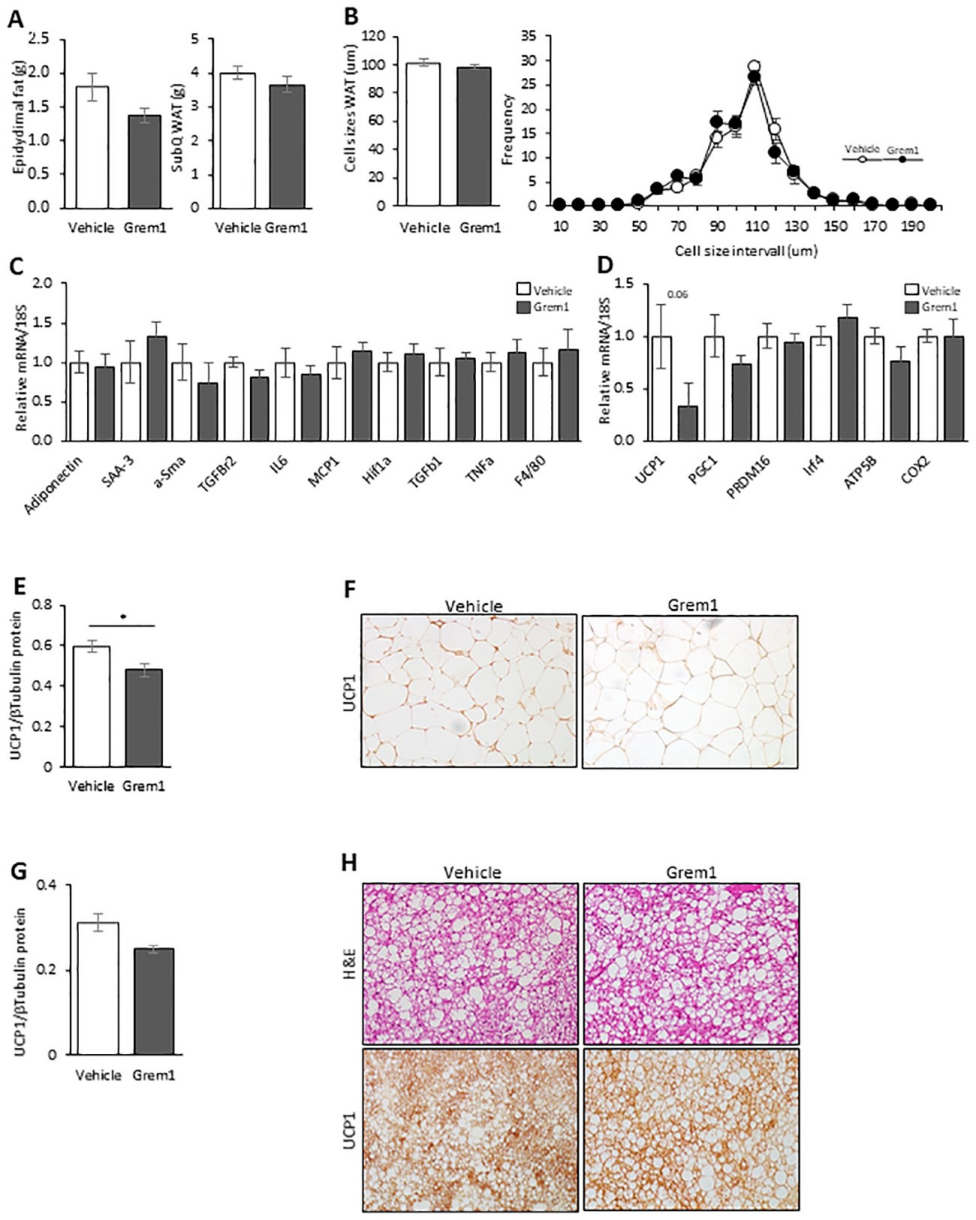
Effect of long-term Gremlin 1 IP injections on WAT and BAT in 38 weeks old male mice (C57BL6/N) fed 60% HFD. (A) WAT weight and (B) subcutaneous adipose cell size. (C and D) qRT-PCR of markers of being, inflammation and fibrosis in SubQ WAT. (E) Graph presenting the UCP1/β-Tubulin protein expression in SubQ WAT using Western blots and band intensities calculated with ImageJ (n = 8 in each group). (F) Representative light microscopy image X20 from SubQ sections showing UCP1 protein expression (n = 6 in each group). (G) Graph presenting the UCP1/β-Tubulin protein in BAT using Western blots and band intensities calculated with ImageJ (n = 4 in each group). (H) Representative light microscopy image X20 from BAT sections showing UCP1 protein expression (n = 4 in each group). Results are means ± S.E.M. Statistical significance was determined using Student’s *t* test (two-tailed). *P<0.05.

Based on our previous studies in human pre-adipocytes showing that GREM1 is an important regulator of pre-adipocyte differentiation and browning [9], we expected to find clear differences between the groups in the adipose tissue as a result of the rGREM1 injections. However, consistent with the lack of effects on body weight in vivo, there were no differences in subcutaneous or epididymal adipose tissue weights (Fig 4A). Furthermore, no differences between the groups were seen for mean subcutaneous adipocyte cell size or size distribution (Fig 4B). In addition, rGREM1 injections did not modify expression levels of several markers for adipogenesis, inflammation and fibrosis in fat (Fig 4C).

We also examined the effect of rGREM1 injections on expression of adipocyte beige markers and the key brown adipocyte marker UCP1. While no effect was seen in any of the beige markers (Fig 4D), rGREM1 injections led to a small, but significant, down-regulation of UCP1 both at mRNA (Fig 4D) and protein levels in the white (Fig 4E and 4F) and brown adipose tissues (Fig 4G and 4H). However, this effect was obviously not sufficient to alter the body weight.

**Fig 5:**
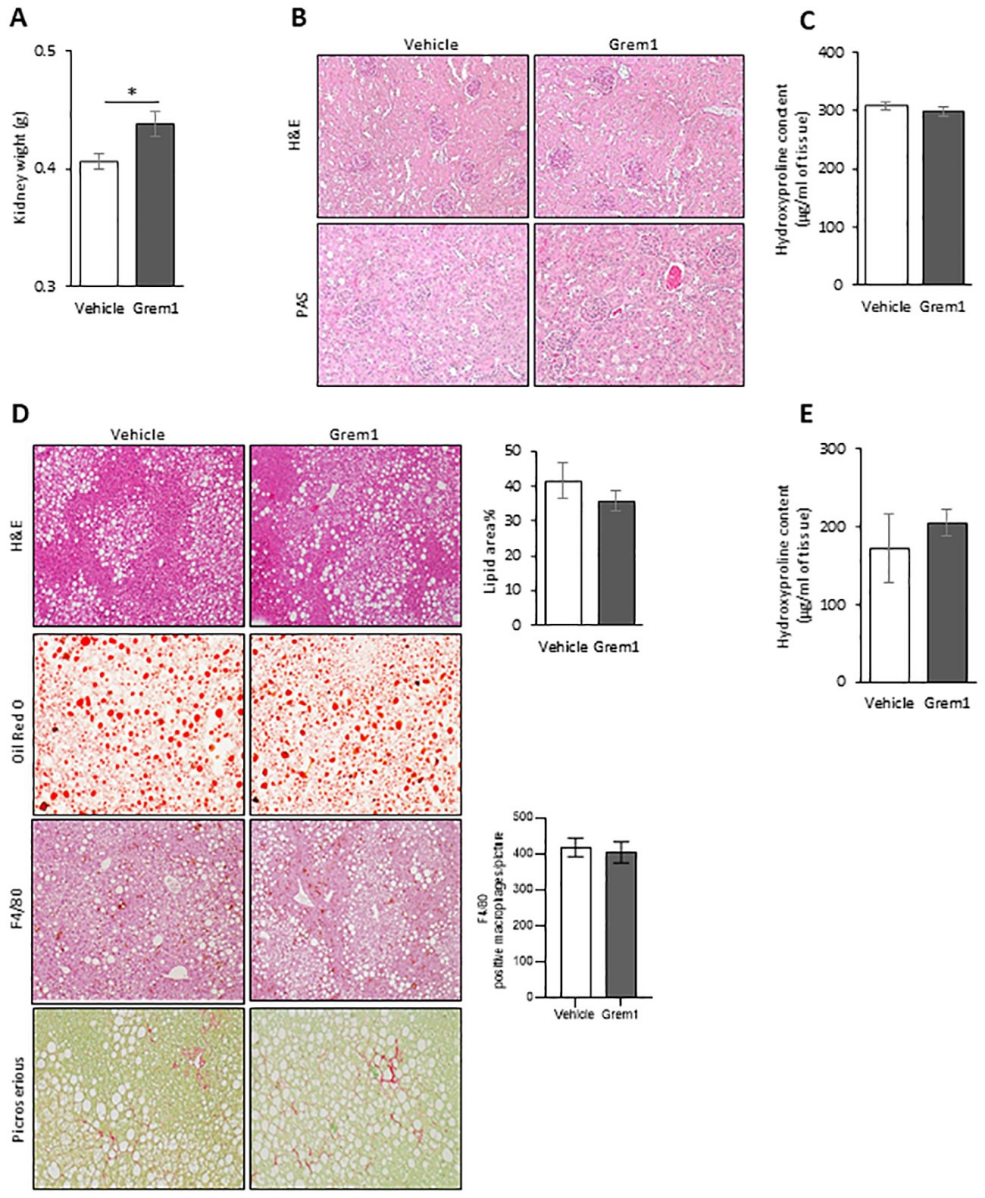
Kidney weight was slightly increased but morphology was not affected by Gremlin 1 IP injections and no effects were seen in liver morphology in 38 weeks old male mice (C57BL6/N) fed 60% HFD. (A) Graph presenting kidney weight. (B) Representative light microscopy image X10 from kidney sections stained with Hematoxylin and Eosin stain (H&E) and Periodic Acid Schiff (PAS), (n = 6 each group). (C) Kidney hydroxy-proline content. (D) Representative light microscopy image from liver stained with H&E, F4/80 (10X for both staining), Oil red O and Picrosirius (20X for both staining) (n = 8 in each group). (E) Hepatic hydroxy-proline content. Results are means ± S.E.M. Statistical significance was determined using Student’s *t* test (two-tailed). *P<0.05.

Since GREMLIN 1 has been reported to regulate kidney development and increase fibrosis in mice [20], we examined kidney weights which were slightly, but significantly, increased by around 10% (Fig 5A). We then examined if injected rGREM1 induced any changes in kidney morphology or hydroxy-proline levels but no such effects were seen (Fig 5B and 5C). We have no explanation for this minor increase in kidney weights and whether this is associated with increased urine production or hyperfiltration since these were not measured.

We also examined if the rGREM1 injections altered liver inflammation, fibrosis or lipid accumulation but no such effects were seen either (Fig 5D and 5E).

Taken together, these data show that *adult* mice are quite unresponsive to increased GREM 1 protein expression in liver cells, a likely consequence of GREM 1 binding to cell proteins and/or is inadequately processed for secretion to the bloodstream (Fig 1A). Since intraperitoneally injected proteins should first reach the liver, the lack of effect seen may also be a consequence of binding to liver cell proteins. Consistent with this, there were also no effects in the liver cells related to increased inflammation or fibrosis. Also repeated intraperitoneal injections did not alter body weight and had only trivial effects on adipose tissue oxidative beige/brown phenotype. Glucose tolerance tended to be improved while early insulin secretion (15 min was the only time of examination) and insulin sensitivity were unchanged. Unchanged insulin sensitivity was also highly unexpected since insulin signalling and action in human cells are antagonized by GREM1 protein in vitro [10]. We, therefore, also validated that GREM1 is a BMP4 antagonist in murine cells and examined potential direct cellular effects of GREM 1 protein on insulin signalling in vitro.

### 3.6. Effect of rGREM 1 on BMP4 pSMAD activation in 3T3-L1 and C2C12 muscle cells

**Fig 6:**
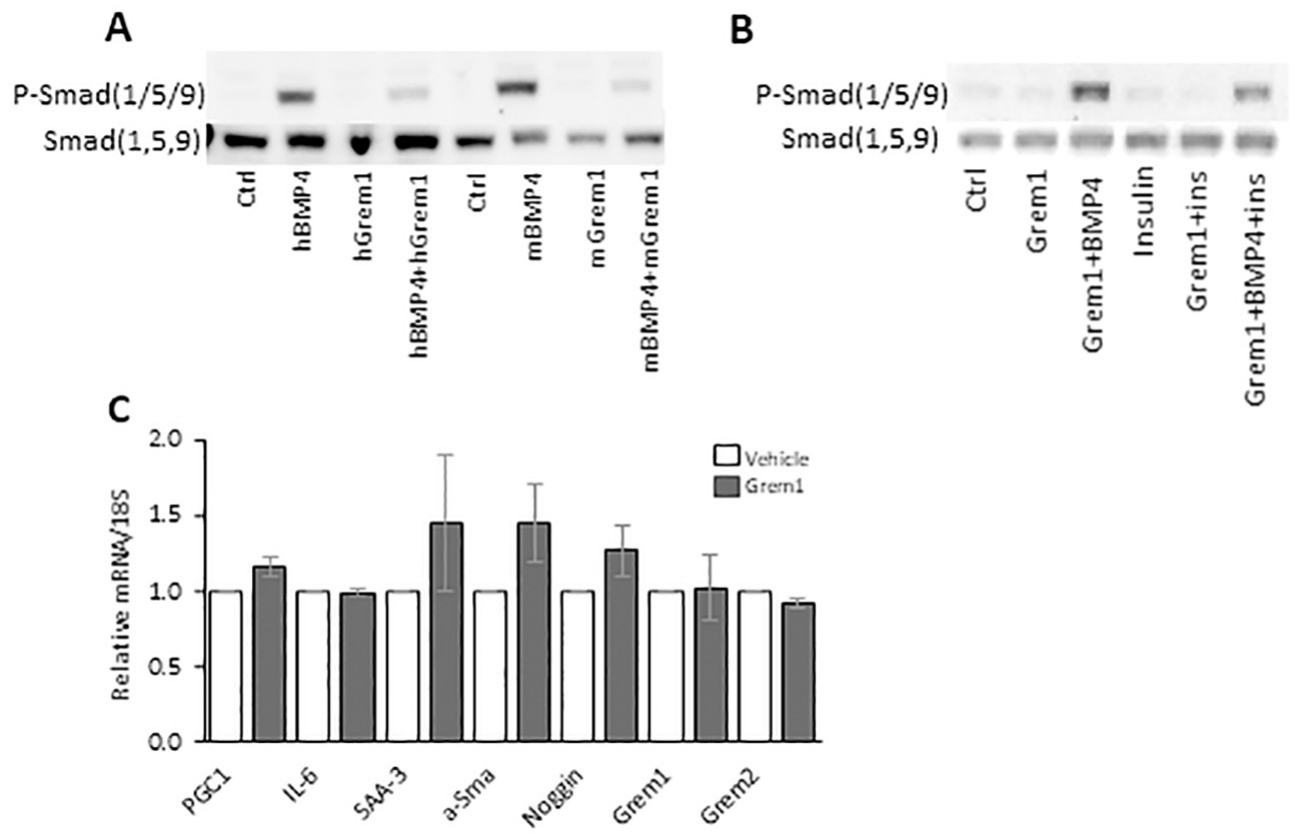
rGremlin 1 inhibits BMP4-induced pSMAD activation but did not change expression of differentiation and inflammation markers in differentiated 3T3/L1 (A) and in C2/C12 cells (B). The cells were first incubated for 3h with rGremlin 1 followed by 1h BMP4 as shown. (A, B) The figures show representative Western blots using antibodies reactive to p-Smad(1,5,9) upper panels and total Smad(1,5,9) lower panels. (C) qRT-PCR of inflammation and fibrosis markers (mRNA/18S) in 3T3/L1 cells (n = 3 separate experiments).

To first validate that GREM 1 is an antagonist of BMP4 in murine adipose precursor cells, we incubated 3T3-L1 and C2C12 cells with recombinant human and murine BMP4 and GREM 1 added to the medium and measured activation of pSMAD 1, 5, 9. Under these conditions, GREM 1 is indeed an inhibitor of both human and murine BMP4 in both 3T3-L1 and C2C12 cells (Fig 6A and 6B). Furthermore, GREM 1 only had small or no effects on markers of adipogenesis and inflammation in differentiated 3T3-L1 cells following 24 hrs incubations (Fig 6C).

### 3.7. No effect of rGREM 1 on insulin-stimulated phospo-Akt (ser 473 and thr 308) activation in murine cells

**Fig 7:**
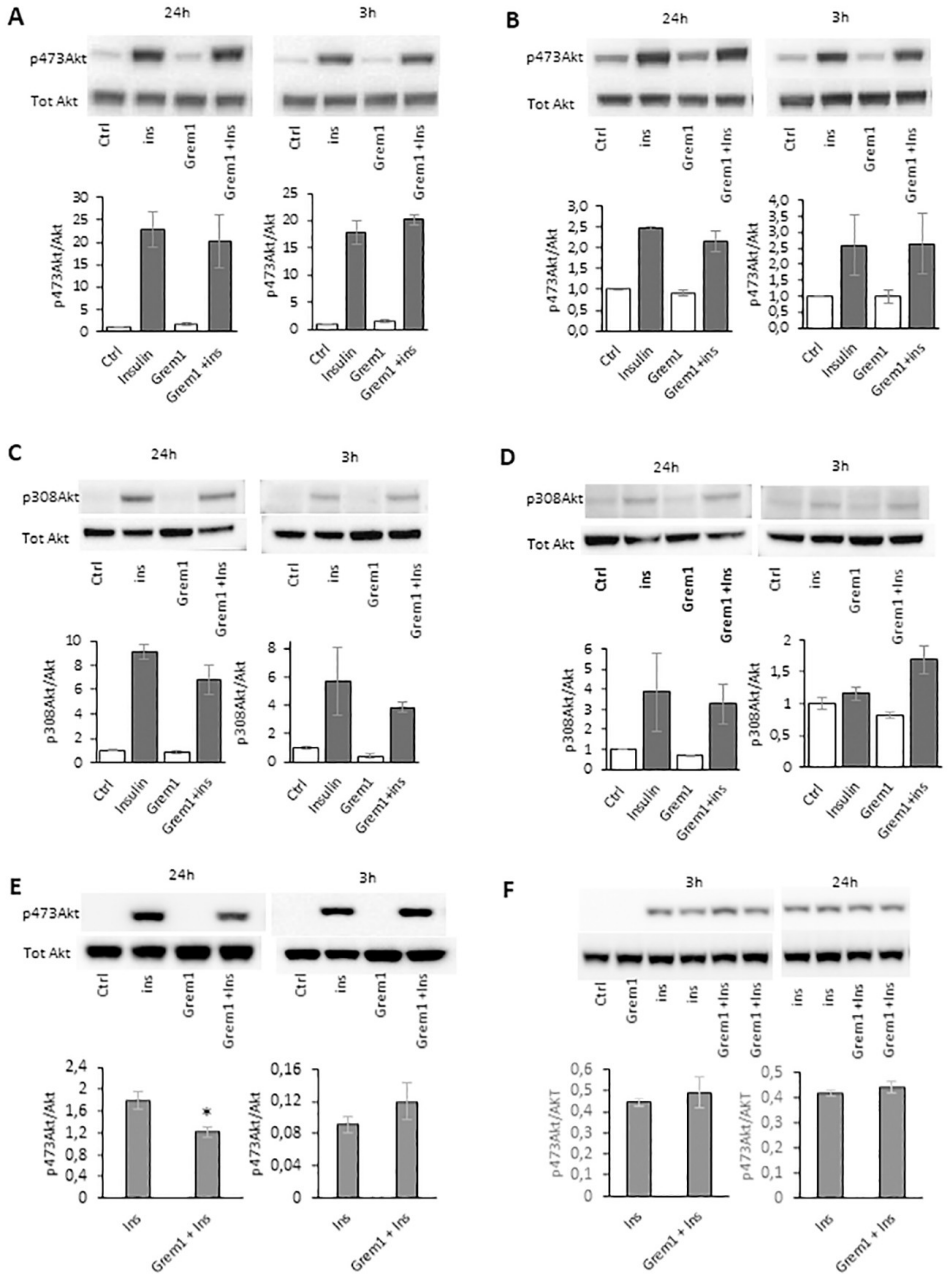
rGremlin 1 did not inhibit insulin signaling in differentiated 3T3/L1 (A, C) or in C2C12 cells (B, D). The figures show representative western blots of cells incubated for 3 h and 24h as shown with/w/o rec-Gremlin1 (400ng/ml). Insulin stimulation was performed with 10nM insulin for 10 minutes. Blots present results with antibodies reactive to p-ser 473 Akt (A, B, E, F) and p-thr 308 Akt (C, D) (upper panels) and total Akt (lower panels). Effect of rGremlin 1 (with validated correct protein folding) on insulin signaling in human IHH liver cells (E) and differentiated 3T3-L1 (F) cells. Band intensities were calculated using ImageJ and plotted on the right as the ratio of p-Akt intensity normalized to total Akt (n = 3 separate experiments). Results are means ± S.E.M. Statistical significance was determined using Student’s *t* test (two-tailed). *P<0.05.

GREM 1 is clearly a BMP4 inhibitor in both murine adipose and muscle cells. We then examined if it also had effects on insulin signalling measured as pAkt activation in these cells. Surprisingly, and in contrast to human adipose, liver and skeletal muscle cells [10], rGREM 1 did not alter insulin-stimulated p-ser 473Akt activation in either 3T3-L1 (Fig 7A) or C2C12 cells (Fig 7B) or p-thr 308 in these cells (Fig 7C and 7D). This was also verified using rGREM 1 with validated correct protein folding, as it inhibited p-ser 473Akt activation in human IHH liver cells (Fig 7E) but not in 3T3-L1 cells (Fig 7F).

## 4. Discussion

DAN/Cerberus family members, including GREMLIN 1, represent the smallest BMP antagonists and have the greatest homology within their central cysteine-rich domain but exhibit significant diversity and low conservation in their termini [1]. GREM 1 is a soluble, extracellular secreted BMP-2, -4 and, to some extent, also BMP -7 antagonist [21] and has important roles in the regulation of embryogenesis and organ development [22, 23]. GREM 1 has three alternative splicing patterns and its post-translational modifications include N-glycosylation and phosphorylation. GREM 1 has both secreted and intracellular forms; secreted GREM 1 is a glycosylated 28 kDa protein [1].

GREM 1 is known to antagonize BMP action by direct binding and inhibiting the BMP-receptor engagement leading to inhibition of canonical BMPs signaling pathways. It is expressed in several tissues and alteration in its expression is linked to several BMP-dependent and independent disorders. For instance, GREMLINS sabotage the mechanism of cancer stem cell differentiation by inhibiting BMPs and some other cell cycle components such as P21. GREM 1 also interacts directly with cell surface members of the Slit protein family [6, 24] and is essential for normal development of kidneys, lung and limb [3, 25].

GREMLIN 1 is also an interesting target in man and in mature animals and is increased in diabetic kidney disease and renal tubule epithelial cell-specific deletion has shown protection against the development of kidney disease [20]. It is also highly expressed, and secreted by, human adipose tissue and is an important regulator of endogenous cell BMP signaling [9]. Our recent study has shown that GREM 1 is increased in the adipose tissue in human obesity and Type 2 diabetes, that it is a secreted antagonist of insulin signaling and action in key metabolic target tissues and that circulating levels tend to be increased in obesity but most prominently in Type 2 diabetes [10]. It is also highly expressed in human liver tissue and further elevated in NAFLD/NASH. However, not much is known about effects of GREM 1 in mature animals in vivo and, specifically, its role in obesity and insulin resistance.

Here we attempted to analyze consequences of increasing circulating GREM 1 levels in vivo in mouse models using two different ways; overexpressing it in the liver with AAV8-*GREM1* vectors or by intraperitoneal injections for several weeks. Intraperitoneal injections of the family member GREMLIN 2 was recently shown to produce effects on inflammation in mice in vivo [2]. However, AAV8-*GREM1* did not produce any clear metabolic consequences in spite of being clearly expressed in the liver cells (Fig 1A). Within the limitations of our assay system, which may not have been sufficiently sensitive to detect small changes in circulating levels, our data suggest that expressed GREM 1 was not adequately processed to be secreted by the liver cells. This apparent lack of cellular processing by mouse liver cells, including secretion and receptor-mediated signaling, is a likely reason for the lack of effect on liver inflammation and fibrosis. Using this gene therapy approach, we have previously shown efficacy in increasing serum BMP4 levels and action as well as protecting the mice from becoming obese by browning the adipose cells and increasing whole-body energy expenditure [9].

Also intraperitoneal injections only produced minor phenotypic consequences. Body weights were unchanged and glucose tolerance was not significantly altered. We could only measure insulin levels at 15 min and did not see any difference but cannot exclude effects of GREM 1 on insulin secretion at other times. There was only a minor reduction in UCP1 protein in white and brown adipose tissues and this was not sufficient to produce any effect on body weight. Recent in vitro studies have suggested that BMP4 can inhibit glucose-stimulated insulin secretion by beta-cells [26] and also inhibit beta cell growth and differentiation [27]. In contrast, other studies have shown that BMP4 is important for normal beta-cell development and that it enhances glucose-stimulated insulin secretion in vivo also in mature animals [28]. In this context, providing increased BMP4 inhibition in these very obese mice, expected to have elevated endogenous BMP4 serum levels [29], may have been expected to reduce insulin secretion but no such effects were seen.

It is also clear that murine cells behave differently from human cells when exposed to similar rec GREM1. We have found that GREM 1 is an antagonist of insulin signaling and action in major human target cells for insulin [10] while no such effect was seen here in murine cells. The reason for this is unclear but the fact that GREM 1 was a potent BMP4 inhibitor also in murine cells differentiate these cellular actions. A potential unifying mechanism is that cellular GREM 1 processing/binding is altered in murine cells, supported by the obvious accumulation in liver cells, and that this is required for antagonizing insulin signaling. Another difference between human and murine cells is the finding that *GREM 1* mRNA levels are low in the adipose tissue in obese mice while levels are high in human tissue. In contrast, *NOGGIN* is high in the adipose tissue in obese mice [16, 17]. Together, these findings suggest that there are species differences in the cellular regulation of BMP 4 signaling and action.

In conclusion, GREM 1 seems to play a major role in regulating BMP4 signaling and action in human metabolic cells while, in similar mouse cells, NOGGIN seems more important. Our data indicate that GREM 1 processing in mature mice, as judged by liver cells, is altered and that it binds to cell proteins rather than being normally cleaved and secreted. We conclude from these data that translating effects of GREMLIN 1 in *mature* mouse models to man, and vice versa, need to be done with caution.

Finally, it should be added that GREM1 is known to exist both as monomers and oligomers with different biological activities [30]. Differences in oligomerization of GREM1 may contribute to different effects observed using recombinant GREM1 protein in vitro and in vivo. Furthermore, it is possible that the apparent lack of secretion of liver-expressed GREM1 with AAV8 is due to improper folding, which could explain the lack of biological effect using AAV8-GREM1 in vivo. However, it cannot account for the differences seen in insulin signaling in human and murine cells in response to recGREM 1 since this difference was also seen with correctly folded recombinant protein. It should also be added that currently available commercial antibodies have a low affinity for GREM 1 protein and cannot be used to safely measure the low levels in serum or in small amounts of tissue/cells. We have used a special antibody with high affinity which, unfortunately, is not produced any more. However, also with this antibody we had problems in our previous study to follow GREM 1 expression in liver cells and had to tag the protein [10]. Thus, there is a great need for better reagents to perform extensive basic work in this area.

## Supporting information

S1 Raw Images (PDF)
