# Supplementary figures and images for: Adult mice are unresponsive to AAV8-Gremlin1 gene therapy targeting the liver
Source: PLoS One. 2021 Feb 19;16(2):e0247300. doi: 10.1371/journal.pone.0247300 (PMC7895349; doi:10.1371/journal.pone.0247300)

Original uncropped blots

Figure 6

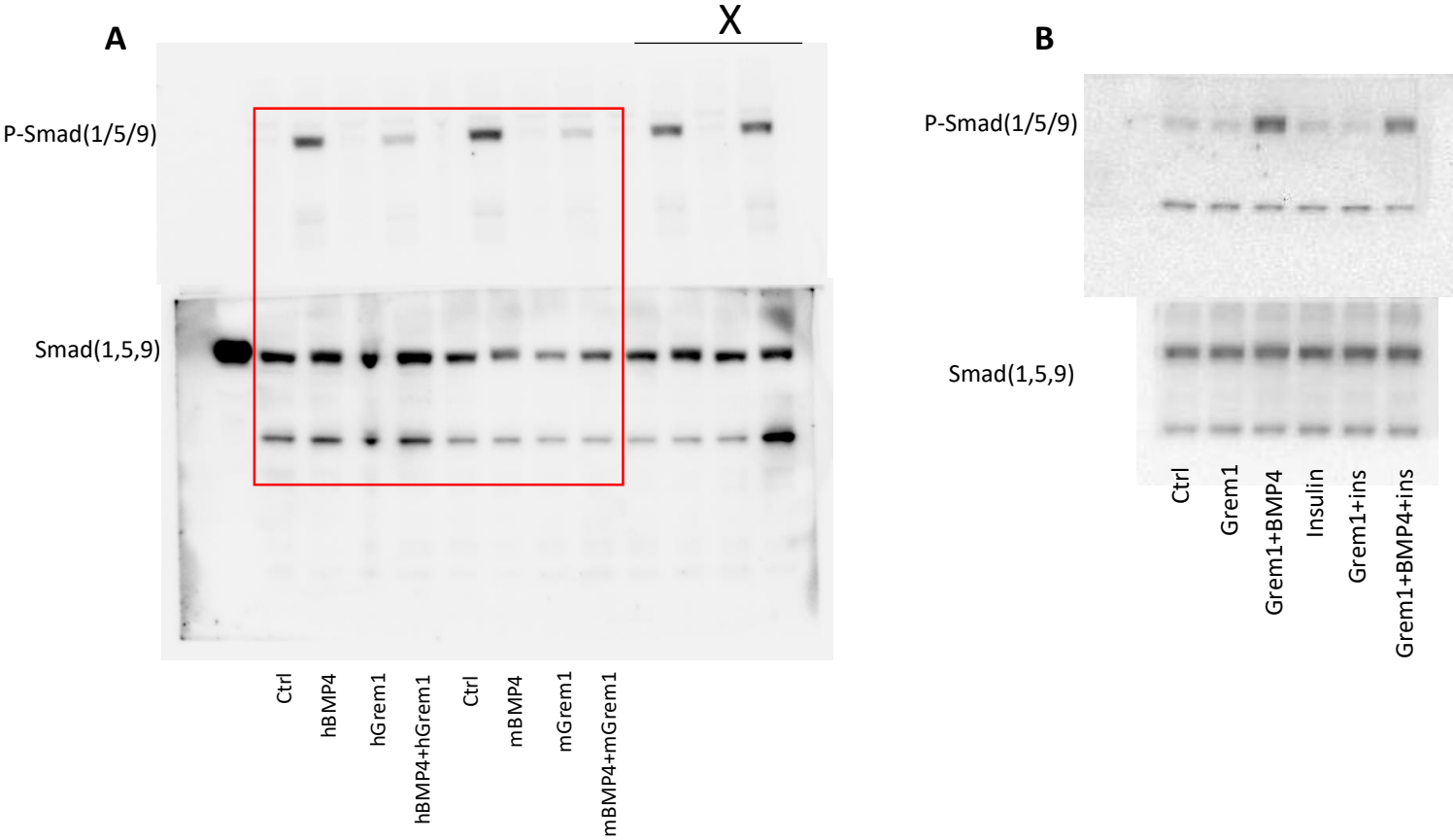

Figure 7

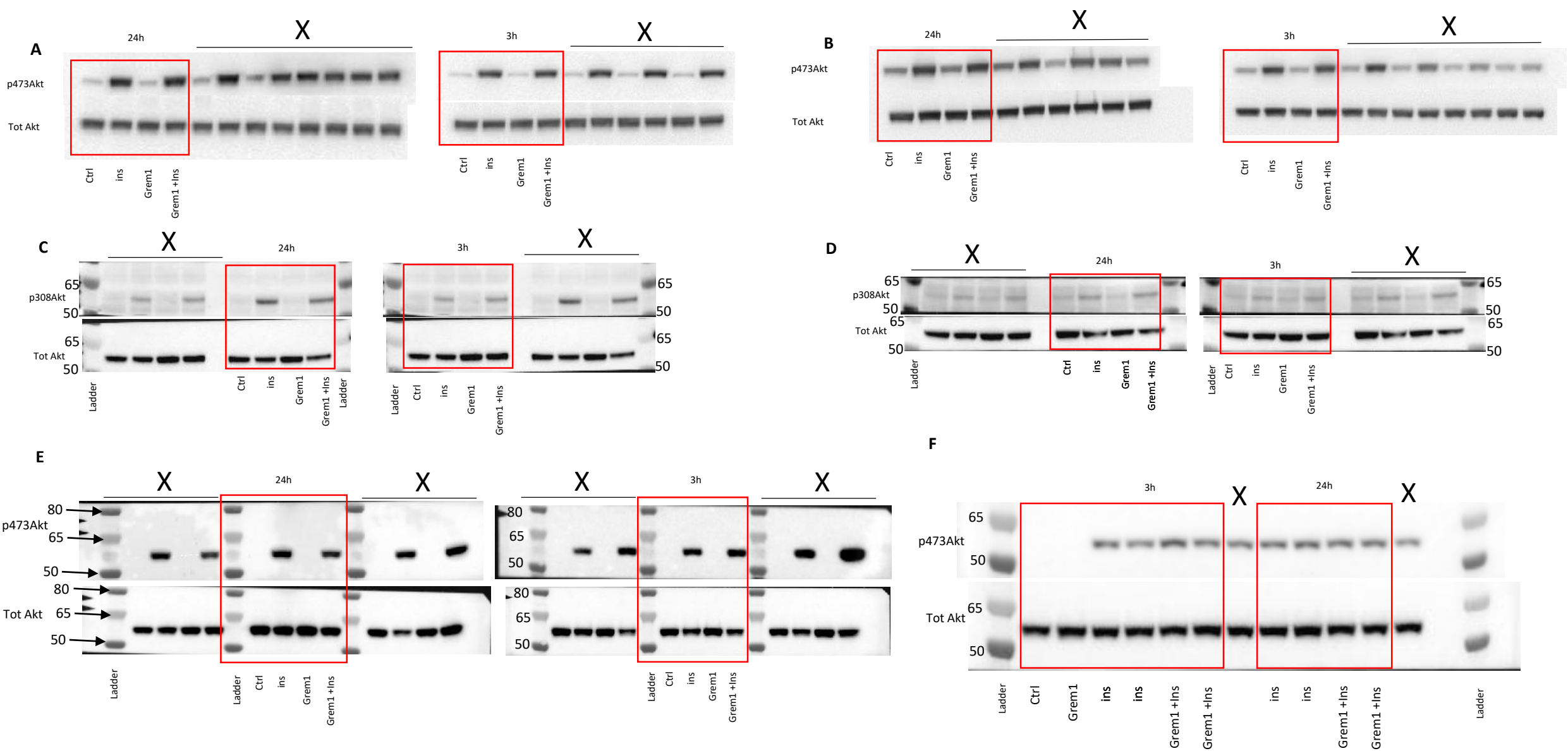

Supplement: S1 Raw Images — (PDF) [file pone.0247300.s001.pdf]
